# Targeting macro- and micro-nutrient regulation of H_2_S signaling for the aging brain

**DOI:** 10.1016/j.neurot.2025.e00638

**Published:** 2025-07-16

**Authors:** Matthew Godwin, Christopher Hine

**Affiliations:** a Department of Cardiovascular and Metabolic Sciences, Cleveland Clinic Lerner Research Institute, Cleveland, OH 44195, USA; b Department of Molecular Medicine, Cleveland Clinic Lerner College of Medicine of Case Western Reserve University School of Medicine, Cleveland, OH 44195, USA

**Keywords:** Hydrogen sulfide (H_2_S), Dietary restriction, Iron, Glioblastoma, Neurodegeneration, Aging

## Abstract

Global growth in aged population demographics is a testament to medical and societal innovations over the past 100 years. However, with advanced age comes declines in various organs and tissues, thus limiting quality of life in one's later years. There are numerous hypotheses for what the driving and causative factors are for biological aging, with each proposing molecular targets and their therapeutic strategies. One such hypothesis is that dysfunctions in cellular and systemic redox homeostasis are to blame, in that aging-related increases in oxidative stress and diminished thiol-mediated protections and signaling activity drive macromolecular damage leading to tissue failure, particularly in the brain. Addressing redox dysfunction has been somewhat a challenge clinically, as antioxidant supplementation has not shown to be universally effective at slowing the aging process. Thus, geroscience interventions that can bolster our endogenous redox machinery may be more effective. In this review, we highlight hydrogen sulfide (H_2_S) and its associated metabolism and gasotransmitter signaling as potent redox mechanisms that can be leveraged via macro- and/or micro-nutrient interventions. Specifically, dietary restriction (DR) and iron status greatly impact the enzymatic and non-enzymatic production, metabolism, and thiol modifications of H_2_S. As both DR and iron status can have profound impacts on redox homeostasis and aging in the brain, we discuss how all these factors are intertwined in glioblastoma (GBM) and neurodegeneration, two of the most significant aging-related disorders of the brain that limit both lifespan and healthspan.

## Introduction

Shifts in population aging demographics over the past 100 years have yielded an ever-growing global segment of those over the age of 65 years [1,2]. This rise can be attributed to advancements and innovations in medicine, public health strategies, sanitary engineering, and cultural evolutions. While this increase in lifespan is largely seen as a positive on both individual and societal scales, it is not without its drawbacks. Namely, advanced life expectancy does not inherently equate to improved healthspan, which is defined as the period of life lived without frailty, terminal illnesses, and/or morbidity [3,4]. The urgency of addressing this issue is highlighted by what is termed the “2030 problem”, in which in the year 2030 an estimated 90 million individuals of the Baby-Boomer generation will reach ages 66 years and above [5]. Thus, improving healthspan in parallel to lifespan is crucial for current and future generations to positively impact quality of life and prevent strains on healthcare systems.

In order to address the unmet needs of healthspan extension, a better understanding of the mechanisms of aging and how they can be leveraged for both preventative as well as reactionary interventions is key. According to López-Otín et al. in their comprehensive review [6], there are twelve hallmarks and/or mechanisms of aging hypothesized, for which they include genomic instability and DNA mutational load, telomere shortening, global and local epigenetic modifications, loss of proteostasis, disabled macroautophagy, disconnected growth- and nutrient-sensing, mitochondrial dysfunction, cellular senescence, stem cell exhaustion and/or lineage bias, altered intercellular communication, chronic inflammation, and dysbiosis [6]. Now, whether these hallmarks are correlative or causative of aging is not entirely understood. Likewise, it is to be determined if these are the actual drivers of aging, or if they are the result of the aging process. Nonetheless, central to many of these hallmarks of aging and a long-held view in aging research originally proposed by Denham Harman [7,8] is that increased cellular production and exposure to reactive oxygen species (ROS) and free radical metabolites drive macromolecular damage of nucleic acids, lipids, and proteins and ultimately the aging process. This free radical theory of aging has also been challenged as well as amended as our understanding of physiological and pathophysiological cellular redox regulation and signaling has evolved over time [[9], [10], [11]]. Thus, a balance between the physiological and pathophysiological roles of sub-physiological or super-physiological ROS and other reactive metabolites is paramount for maintaining health, and in particular, neurological function [[12], [13], [14], [15]]. As brain- and neurological-specific declines in cognition and tumor suppression are recognized as some of the most severe and irreversible casualties of aging, it is not surprising a great deal of effort has already been committed to understanding the role of redox balance in these disorders [[16], [17], [18], [19], [20], [21], [22], [23]].

Addressing these redox and reactive metabolite dysfunctions has been somewhat a challenge clinically, as antioxidant supplementation in the form of oral, intravenous, and/or topical applications have not shown to be universally effective at slowing the aging process [[24], [25], [26]]. Furthermore, aging-related neurodegenerative disorders such as Alzheimer's [26,27] and Parkinson's Diseases [28,29] and the aging-related neuro-oncological cancer glioblastoma (GBM) [30,31] have not benefited greatly from antioxidant treatments. Thus, geroscience interventions that aim to slow the aging process that can bolster and balance our endogenous redox machinery may be more effective for clinical translation of neurological disorders.

In this review, we highlight hydrogen sulfide (H_2_S) and its associated metabolism and gasotransmitter persulfide signaling [32,33] as central pathways in geroscience approaches that act as potent redox modifiers that can be leveraged via macro- and/or micro-nutrient interventions [34]. Specifically, dietary restriction (DR) and iron status impact the enzymatic and non-enzymatic production and metabolism of H_2_S. As both DR and iron status can have profound impacts on redox homeostasis and aging in the brain, we discuss how all these factors are intertwined in glioblastoma and neurodegeneration, two of the most significant aging-related disorders of the brain that limit both lifespan and healthspan.

### Nutrient modification of hydrogen sulfide production and signaling

Geroscience is an approach that identifies drivers of the aging process and targets the basic biology of aging as the main therapeutic target to provide delays in the onset and/or severity of multiple aging-related disorders including neurological decline, inflammation, and cancer [35,36]. Effective geroscience approaches that have been shown to improve lifespan and/or healthspan in model organisms and experimental laboratory studies include: 1) Various forms of dietary restriction (DR) without malnutrition such as caloric restriction (CR), intermittent fasting (IF), and methionine restriction (MetR) [34,37]; 2) Endocrine manipulation in which growth hormone (GH), thyroid hormone (TH), and/or insulin-like growth factor-1 (IGF-1) activity are diminished [[38], [39], [40]]; and 3) Pharmacological interventions, such as rapamycin and metformin, that are largely studied and/or validated by the National Institutes of Health/National Institute on Aging Interventions Testing Program [41,42].

While these three intervention types first appear to be disparate form each other, investigations into commons pathways and/or molecular phenomena shared by all three may identify crucial pathways to target. This task is highlighted in work by the Gladyshev lab, in which hepatic gene expression profiles were compared from 3 different lifespan extending DR interventions (CR, IF, and MetR), 8 different lifespan extending genetic/endocrine manipulations, and 4 different lifespan extending pharmacological interventions. They determined a common denominator shared by all three types was enhanced hepatic expression of cystathionine γ-lyase, also referred to as cystathionase and abbreviated as CGL, CTH, or CSE [43]. CGL is an integral pyridoxal phosphate-dependent enzyme in the transsulfuration pathway where the amino acid l-cysteine is ultimately produced from the essential amino acid l-methionine. In addition to this canonical pathway, CGL participates in α,β-elimination or β-replacement of the thiol groups from cysteine and homocysteine to produce hydrogen sulfide (H_2_S) [44,45].

So while the increased CGL expression shared amongst these interventions was in the liver, due to the liver being one of the strongest producers of H_2_S [[46], [47], [48]] and the circulating gasotransmitter activities of H_2_S [49,50], it is reasonable to hypothesize systemic alterations in sulfide signaling occur under these geroscience interventions. Evidence for this rests in previous work showing CR and IF inducing CGL-dependent enhancements in protein persulfidation in multiple tissues in the body, and importantly in the brain, despite only having increased H_2_S production in the liver and kidney [47,51]. H_2_S is also produced in mammalian tissues via the enzymatic activities of cystathionine β-synthase (*aka* CBS) and the mitochondrial 3-mercaptopyruvate sulfurtransferase (*aka* 3-MST) [52]. Between CGL, CBS, and 3-MST lies differences in tissue specificity for their expression as well as their sub-cellular localization [53,54]. Non-enzymatic H_2_S production can also be catalyzed by coordinated breakdown of cysteine via iron and vitamin B_6_ [55]. Outside of mammalian tissues, the microbiota residing in our bodies can generate significant amounts of H_2_S [[56], [57], [58]], sometimes even surpassing our own endogenous cellular production [59].

The chemical oxidation state of −2 for the sulfur in H_2_S is indicative of it serving as a reducing and antioxidant agent allowing H_2_S to perform several important non-mutually exclusive biochemical reactions and processes *in vivo*. These biochemical activities include alterations of iron-sulfur and heme centers, protective protein posttranslational modifications and signaling on reactive cysteine residues through persulfidation, and mitochondrial electron transfer through sulfide quinone oxidoreductase (SQOR) leading to ATP production [[60], [61], [62], [63]].

Aging-related declines in tissue-specific as well as systemic H_2_S levels and/or production were first reported in the early to mid-2000's. Under disease conditions, and specifically Alzheimer's Disease, brain and plasma were found to have decreased sulfide content [64]. In non-disease states, Predmore et al. reported in 2010 that aortic H_2_S concentrations, and contractile responses to H_2_S, decrease with advanced age [65]. In more recent studies, the Filipovic group showed losses in protein persulfidation and expression of all three H_2_S generating enzymes in the brains, hearts, and livers of aged rats [32]. In human cells and plasma taken from healthy individuals, aging-related declines in persulfidation and/or sulfide were detected at ages as early as the 30s and 40s [32,59].

Both micro- and macronutrients have demonstrated modulatory roles in H_2_S production, signaling, as well as in inflammation, capable of both exacerbating and attenuating neuroinflammation. Micronutrients such as zinc and selenium act indirectly as antioxidants by being cofactors in antioxidant enzymes superoxide dismutase and glutathione peroxidase, respectively [66]. Vitamin D upregulates the transcription factor NRF2 which activates several antioxidant related genes [67]. Fats and lipids have been shown to exhibit both inhibitory and stimulatory effects on inflammation depending on the species of lipid. Omega-3 unsaturated fats reduce IL-6/TNF- α and have an inhibitory effect on inflammation [68]. In contrast, saturated fats upregulate inflammation by increasing ROS and activating TLR4, promoting pro inflammatory pathways including MAPK, and NF-κB [69]. In this context, H_2_S is highly neuroprotective by modulating NF-κB [70], upregulating NRF2 [71], and improving mitochondrial function [72,73].

Thus, as enhanced H_2_S appears to be central to extend lifespan and healthspan [1,32,34,43,[74], [75], [76], [77], [78], [79], [80]] yet its diminished presence is at the very least is correlative of shortened lifespan and healthspan [[81], [82], [83]], it becomes essential that we devise interventions to boost H_2_S during aging. Numerous additional studies over the past 15 years have demonstrated diet and nutrient availability as modifiable factors impacting H_2_S production and its downstream signaling via protein persulfidation. Many of these we have previously highlighted in depth here [1], here [34], and here [84]. In the following sections, we focus on two important parts of brain aging, these being neurodegeneration and the primary malignancy glioblastoma, and discuss the impact of dietary- and nutrient-availability on their development, progression, and treatment in the context of redox alterations and sulfide biology.

### Neurodegeneration, dietary restriction, and hydrogen sulfide

Deficiencies in endogenous enzymatic H_2_S production are associated with a number of aging-related neurodegenerative disorders including Huntington's [85], Alzheimer's [72] and Parkinson's [86] disease in rodent and cellular models. Importantly, the field of endogenous H_2_S production and signaling serving physiological functions began in the brain, with the first report of the significance of endogenous enzymatic production of H_2_S is that it serves as a neuromodulator [87]. Since then, the field of H_2_S in neuromodulation and neurodegeneration has expanded due in part of the efforts from the labs of Synder [88], Paul [89], Kimura [90] and many others. With such a plethora of H_2_S-based molecular and pathophysiological discoveries made over the past several decades, how do we harness this information for the treatment of neurodegenerative diseases?

As DR and its many forms have been studied since the early 1900's to address cancer [91], longevity [92], stress resistance [93,94], and metabolic fitness [95,96], it also has a storied history in neurocognitive and neurodegenerative studies [97,98]. In the settings of both experimental laboratory animal models as well as in human clinical trials, DR in its many forms including CR and IF has shown to be neuroprotective, improve cognition during normal aging [51,[99], [100], [101]] as well as in specific neurodegenerative diseases [[102], [103], [104]]. Much of this work has been championed by the lab of Mark Mattson [105,106], with molecular mechanisms proposed that include hippocampal neurogenesis, reduced inflammation, neuro vascular improvement, and enhanced synaptic plasticity under DR [107,108]. Conversely, high fat or Western style diets worsened both normal and pathological neurological aging [109,110].

Disulfide and sulfide modifications are crucial to protein folding and sulfur amino acid (SAA) sensing and prevention of oxidative stress. Lack of sulfide amino acids in the diet can be sensed in a GCN2- and ATF4-dependent manner, boosting H_2_S production concurrent with the integrated stress response [[111], [112], [113], [114]]. Cysteine disulfide bonds regulate protein activity and folding under oxidative stress by acting as a reversible redox switch. In the context of aging, endogenously produced H_2_S causes proteins to undergo persulfidation. Persulfidation associated with dietary restriction has been suggested to promote lifespan in rodents and C. elegans [32,47]. The proposed mechanism by which persulfidation is protective is by protecting thiol groups from irreversible oxidative stress as well as modifying protein structure and function impacting metabolic and protein homeostasis pathways [50,115].

**Fig. 1:**
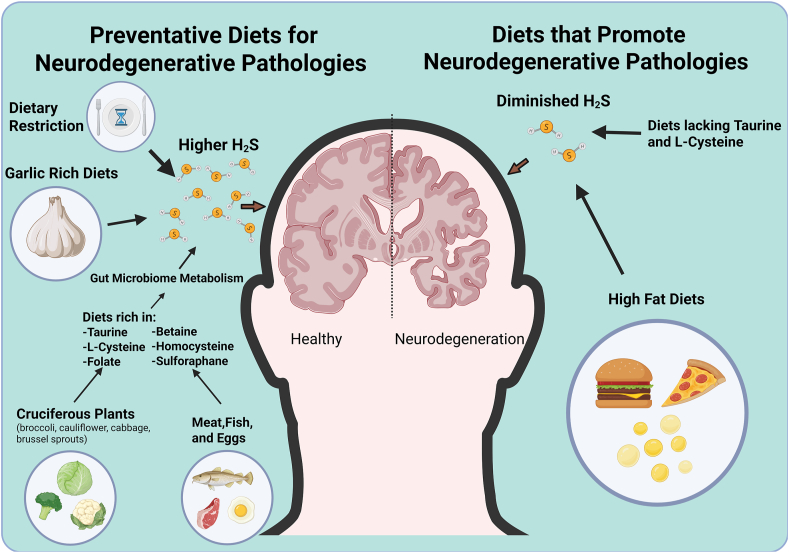
**Dietary impacts of neuroprotective H_2_S.** Hydrogen sulfide (H_2_S) serves as a potent neuromodulator and neuroprotectant that may protect against neurodegeneration. On the left, diets and dietary interventions that may boost neuroprotective H_2_S are detailed. On the right, diets that may suppress neuroprotective H_2_S.

As there is a decrease in brain H_2_S production and/or persulfidation in aging and in neurodegenerative diseases [32,89,116], and DR can boost H_2_S and persulfidation in the brain [32,47] concurrent with improved neurocognitive endpoints as highlighted above, it raises the question if the mechanisms of action for which DR protects against neurodegeneration are partially through boosting local or systemic H_2_S and persulfidation. Correlative evidence for enhanced H_2_S production and brain persulfidation under both CR and IF come in aged male mice [47,51,117]. As DR to boost H_2_S for the treatment of neurodegeneration may be difficult to widely implement in human populations, it is suggested other forms of administering small molecules and sulfide-containing drugs and nutrients to boost H_2_S levels should also be investigated [118]. The sulfur containing DADS and DATS molecules of garlic have been shown to elevate circulating H_2_S levels, which would potentially cross the blood brain barrier [[119], [120], [121]]. It is also hypothesized that a diet rich in sulfur containing nutrients and/or cofactors involved in sulfur amino acid metabolism such as taurine, cysteine, folate, vitamin B_12_, vitamin B_6_, and betaine may lessen risk for Alzheimer's disease by boosting brain synthesis of hydrogen sulfide [122]. It may also be the gut microbiota acting on these sulfur-containing small molecules and nutrients to produce H_2_S that enters the host circulation, and thus leveraging our commensal's strong ability to produce H_2_S to usher in novel treatment opportunities. Depiction of these hypotheses leveraging diet and nutrients to impact neuroprotective H_2_S are shown in Fig. 1.

### Glioblastoma, diet, and hydrogen sulfide

Primary glioblastoma (GBM) with wild-type isocitrate dehydrogenase (wtIDH) represents a large majority of aging-related GBM diagnoses. Despite aggressive therapy consisting of combinatorial surgery, radiation, and chemotherapy, GBM remains uniformly lethal. Prognosis for patients with GBM is dismal, with a median survival of 14–16 months and a 5-year survival rate of less than 3 ​% [[123], [124], [125]]. While GBM can arise at any age, there is a dramatic enrichment among patients ≥65 years of age. Coincident among this population is overall survival of <6 months and fewer long-term survivors [126]. This is consistent with the hypothesis that advanced age increases systemic, tumor-intrinsic, and/or tumor-microenvironmental factors contributing to and/or decreases factors suppressing the onset, progression, and immune system evasion of GBM. These bleak statistics highlight the need to discover aging-related mechanisms accounting for the poorer prognosis among older adults with GBM to improve treatment approaches and outcomes.

Although decreased H_2_S is strongly associated with and well-studied in the first three of these aging-related disorders, it is less understood in the context of cancer. H_2_S is pro-tumorigenic in colorectal [45,127] and ovarian [128] cancer, primarily due to its pro-angiogenesis attributes [127,129]. Conversely, H_2_S, particularly when delivered exogenously, acts to suppress tumorigenesis in numerous other cancer cell types *in vitro* and *in vivo* [130], due to its alterations of cancer cell metabolism and/or sensitization of cells to apoptosis [131,132]. Moreover, there is emerging evidence that H_2_S can alter tumor immunity through suppression of the GBM-permissive indoleamine 2,3 dioxygenase 1 (IDO1), an enzyme that normally enhances immune suppression in the tumor microenvironment [133]. Thus, developing and utilizing safe H_2_S-related therapies will serve as anti-GBM treatments in themselves and/or in combination with standards of care.

Little is known regarding endogenous H_2_S levels and related persulfidation signaling in GBM, or the function of H_2_S in GBM. In our initial interrogation of patient databases, we found patients with low CBS and MPST mRNA in GBM biopsies have significantly poorer prognosis [134]. While this represents gene expression on an mRNA level and not protein levels nor enzymatic activity (specifically that of CGL), it offers a clinical basis supporting our hypothesis that endogenously produced H_2_S acts as a GBM suppressor that is modified by age and/or oncologic disease status.

To test this hypothesis, we previously interrogated H_2_S production and persulfidation signaling in GBM samples from male and female patients and compared to non-tumor control brain tissue. GBM samples had reduced H_2_S production, and as detected via a multi-step biotin-thiol assay combined with LC/MS-MS proteomics analysis approach, a substantial reduction in protein persulfidation in GBM biopsies [134], suggesting H_2_S as a non-genetic, potentially replaceable GBM tumor suppressor.

How can diet play a role in aging-related GBM? We previously showed consumption of an obesogenic and aging-accelerating [135,136] high fat diet (HFD) modifies the trajectory of GBM. Mice fed a high fat diet (HFD) succumbed to disease more rapidly compared to mice fed control diet [134], with the HFD also enhancing cancer stem cell properties [134]. It should be stressed that HFD has pleiotropic effects, including suppression of H_2_S production and sulfide availability [137]. In our models, HFD reduced H_2_S production and protein persulfidation in the brains of tumor-bearing mice on HFD compared to control chow diet [134]. While these initial data showed a correlation between GBM status and decreased H_2_S production capacity and/or persulfidation signaling, they do not directly provide evidence for the tumor suppressive effect of H_2_S on GBM. To directly test if an increase in H_2_S production suppresses GBM aggression, we administered exogenous H_2_S through intratumor injection of sodium hydrosulfide (NaHS), a quick-releasing and short-lived sulfide salt [130], for 10-days in GBM tumor bearing HFD fed mice. Direct NaHS treatment resulted in decreased tumor volume and reversed HFD-induced cancer stem cell phenotypes in tumors and surrounding microenvironment [134]. These initial data provide evidence that indirect H_2_S suppression accelerates GBM growth and self-renewal, while direct boosting and application of H_2_S attenuates GBM growth and self-renewal.

**Fig. 2:**
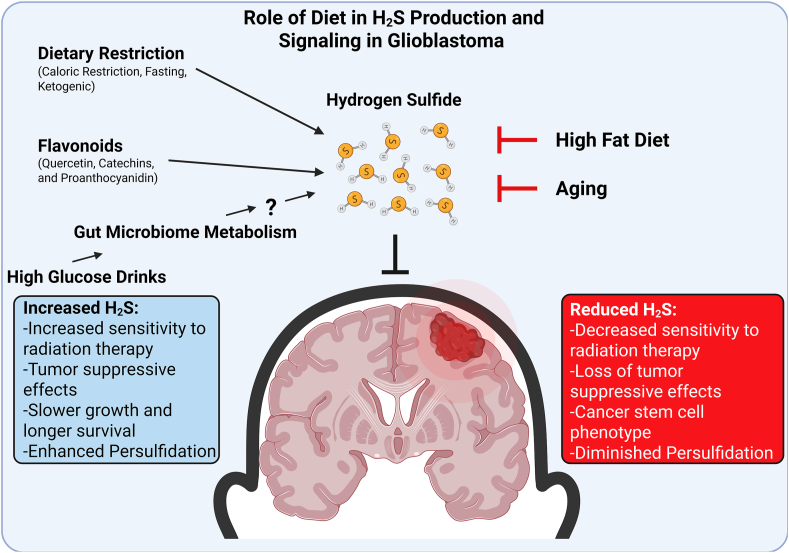
**Diet and Age impact Tumor Suppressive Hydrogen Sulfide in Glioblastoma.** Hydrogen sulfide (H_2_S) acts as a tumor suppressor in a number of cancers, including the neurological cancer glioblastoma. The tumor suppressive ability of H^2^S may rest in how sulfide is metabolized in a cancer-cell intrinsic manner. On the left, diets and dietary interventions that may boost tumor suppressive H_2_S. On the right, diets and aging factors that may suppress tumor suppressive H_2_S.

However, the function of H_2_S in cancer varies depending upon its context of cell type and the sulfide metabolizing machinery present. While we and others have shown H_2_S functions as a tumor suppressor in glioblastoma [134,138,139] (Fig. 2), others have shown the activity of CGL and/or H_2_S production also promote glioblastoma invasion in different models while also limiting overall tumor burden [22,140,141]. Thus, there appears to be a paradox or at the least much to be discovered to explain these differential study outcomes, such as future studies aimed not just on the H_2_S production side of GBM, but also on H_2_S oxidation and removal by the mitochondrial SQOR system [142,143]. Therefore, possibly enhancing H_2_S production along with diminishing its mitochondrial removal may be a promising method for GBM treatment. Having increased insight into this paradox is crucial, as once it is resolved on how to have H_2_S be solely tumor suppressive in GBM, applications of H_2_S to complement standards of care, such as radio- and chemo-therapies, will be feasible and efficacious. Previous studies have shown H_2_S and/or H_2_S donor molecules to sensitize various tumor cells of cervix and breast origin to radiotherapy and/or chemotherapy [144], while studies in other cancer types found H_2_S to invoke resistance [145,146]. Notably, boosting endogenous H_2_S or supplementing with exogenous H_2_S donors was shown to be radioprotective to non-cancerous cells such as in the bone marrow [147]. Additionally, future studies are needed to examine additional geroscience interventions such as DR and growth hormone and thyroid hormone modulation for boosting endogenous H_2_S production and signaling in the treatment of GBM concurrent with standard of care.

### Iron, thiol, and hydrogen sulfide interactions

A common factor in neurodegenerative diseases and in GBM is the accumulation of iron throughout the central nervous system and/or the tumor microenvironment [[148], [149], [150], [151]]. While iron is essential for cellular and organismal health via its roles in oxygen transport, respiration, and cytochrome mediated detoxification [[151], [152], [153]], it also possesses a strong ability to form cytotoxic reactive oxygen and metabolite species (ROMS) as a catalyst and/or reactant in the Fenton reaction [154]. It is hypothesized the increase in ROMS by iron overload drive neurodegeneration and GBM initiation and progression by damaging proteins, DNA, and lipids while inducing the pathological formation of stereotypical protein aggregates found in a number of neurodegenerative diseases [155,156]. Current clinical therapies targeting iron accumulation via metal chelation [157,158], or ROMS via antioxidants [159], have been met with mixed results. Therefore, better characterizing, controlling, and/or taking advantage of iron catalyzed reactions in the brain remain as challenges and opportunities for treating aging-related neurodegenerative diseases. Thus, we need a better understanding of the chemical mechanisms and physiological consequences of iron dysregulation in the brain.

The role of iron and thiol groups are crucial in biological systems because of their involvement in redox processes. Iron exists in different oxidation states, primarily ferrous (Fe^2+^) and ferric (Fe^3+^), aiding in its role in electron transfer that are essential for various metabolic, signaling, and biochemical processes in the cell [160]. The redox versatility of iron enables it to serve as a cofactor for many enzymes, particularly those involved in oxygen transport, energy metabolism [161] and bioactivation [[162], [163], [164], [165], [166]]. Thiol groups on proteins and peptides, which predominantly involve a sulfur covalently bonded to hydrogen on cysteine and methionine residues, are extremely important in biological systems because they have nucleophilic properties, enabling them to participate in redox reactions and form disulfide bonds that help to stabilize the structures of proteins [167]. The reactivity of thiols is mainly attributed to the presence of sulfur with its many different oxidation states, ranging from −2 to +6, forming disulfides or sulfenic acids hence altering cellular redox states [167].

Iron-thiol interactions in biological systems are crucial for maintaining redox homeostasis, which is vital for cells to function and survive. Iron-thiol complexes modulate the availability of reactive oxygen species (ROS), which are byproducts of cellular metabolism that can cause oxidative stress if not properly regulated [168]. The Fenton reaction, involving iron and hydrogen peroxide, generates hydroxyl radicals, a potent form of ROS that damages cellular components, including lipids, proteins, and DNA [169]. Conversely, thiols act as antioxidants, neutralizing ROS and protecting cells from oxidative damage [167]. Thus, the balance between iron and thiol levels is essential for cellular health. Dysregulation of these interactions can lead to various pathophysiological conditions, including neurodegenerative diseases, cancer, and cardiovascular disorders [170].

This section of the review aims to provide an overview of iron-thiol reactions in pathophysiology, exploring the chemical and biological significance of these interactions, and includes several key areas: **A**) Iron chemistry and redox reactions, describing the fundamental principles governing iron's behavior in biological systems; **B**) Thiol chemistry in biology, focusing on the roles of thiols in redox reactions and their interactions with iron, **C**) Mechanisms of iron-thiol interactions and their implications for pathophysiology, including how these reactions influence enzymatic activity and contribute to disease conditions; **D**) Physiological roles of iron-thiol reactions, emphasizing their importance in cellular signaling and metabolic pathways, **E**) Pathophysiological implications of iron-thiol dysregulation, detailing how imbalances can lead to oxidative stress and disease, and **F**) Therapeutic strategies targeting iron-thiol interactions, including the potential for iron chelation therapy and antioxidant supplementation.

Together, this section and respective sub-sections will highlight the interplay between iron and thiol groups as fundamental aspects of biological chemistry with profound implications for health and disease. Understanding these interactions will be vital to inform the development of novel therapeutic strategies aimed at mitigating the effects of iron-thiol dysregulation in various neurodegenerative and neurooncological contexts.

A) Iron Chemistry and Redox Reactions:

**Iron in Biological Systems:** Iron is an essential trace element and micronutrient in biological systems, playing a crucial role in various cellular processes. In a biological context, iron exists in several forms, primarily as heme, non-heme iron, and iron-sulfur clusters. Heme is an iron-containing compound integral to oxygen transport and storage, found in hemoglobin and myoglobin. Non-heme iron is involved in various enzymatic reactions, while iron-sulfur clusters (ISCs) serve as cofactors in several proteins, facilitating electron transfer and redox reactions necessary for cellular metabolism [[171], [172], [173]]. The dynamic balance of these different forms of iron is vital for maintaining cellular homeostasis, allowing it to participate in redox reactions that are critical for energy production and metabolic regulation [174,175].

**Fig. 3:**
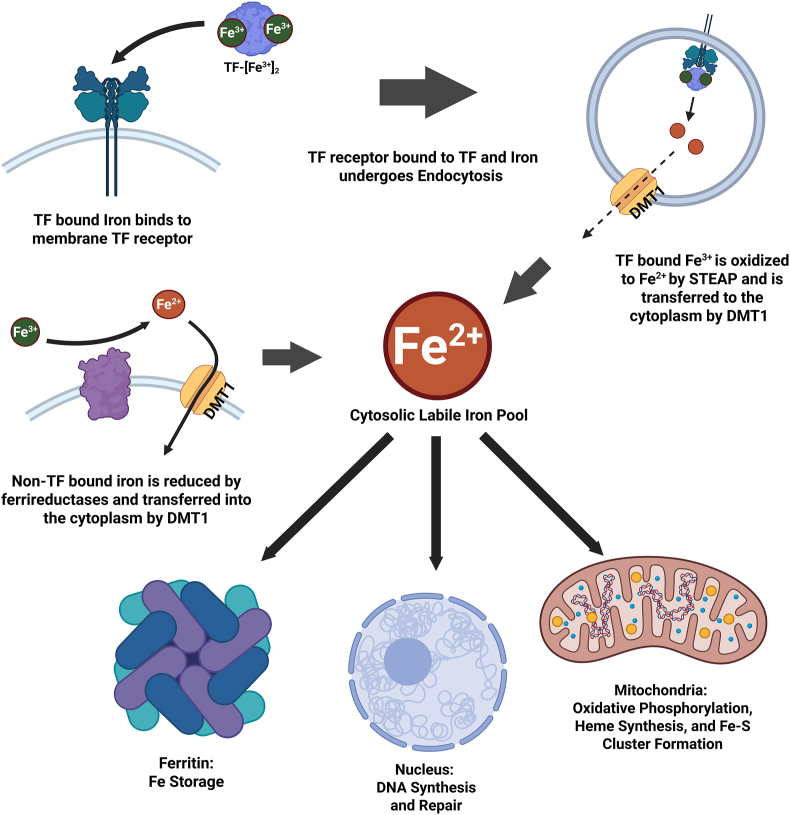
**Overview of Iron Trafficking and Compartmentalization.** Iron enters the cell either through transferrin (TF)-mediated endocytosis or directly through DMT1. Cytosolic iron is then transferred to the mitochondria for cellular respiration, the nucleus for DNA synthesis and repair, or stored in ferritin.

Iron homeostasis is tightly regulated across different cellular compartments to perform essential functions and moderate potential toxicity. Cellular iron transport involves specific uptake and ferric reduction, intracellular distribution, storage, and utilization. Iron enters the cytoplasm either through transferrin receptor-mediated endocytosis or directly facilitated by divalent metal transporter 1 (DMT1) following the reduction of non-TF iron by ferrireductases where it is released into the cytosolic labile iron pool to be distributed within the cell based on demand [[176], [177], [178], [179]]. Mitochondria are the primary consumers of intracellular iron for use during cellular respiration and metabolism [180]. Iron is also directed to the nucleus to assist in DNA synthesis and repair mechanisms [181]. Excess iron is stored in ferritin to prevent iron overload and oxidative damage [182]. This integrated process of iron homeostasis is illustrated in Fig. 3.

The complex interaction between iron and ROS is a necessary double-edged sword; while iron is essential for many biological functions, detrimental oxidative stress can damage cells and cause diseases. Cells have evolved complex mechanisms to regulate iron homeostasis and mitigate oxidative damage, including the expression of iron regulatory proteins and the synthesis of antioxidants [174,175]. This underscores the importance of the homeostasis between iron's beneficial roles and its potential for harm in the context of metabolism and disease.

**Overview of Redox Reactions Involving Iron:** The Fenton and Haber-Weiss reactions are pivotal in understanding the redox chemistry of iron and its implications in biological systems. The Fenton reaction illustrates how ferrous iron catalyzes the decomposition of hydrogen peroxide, generating reactive hydroxyl radicals [183,184]. These radicals cause lipid peroxidation, protein oxidation, and DNA damage. This leads to cellular dysfunction and contributes to the pathogenesis of various diseases and other mitochondrial disorders [173,185]. The Haber-Weiss reaction amplifies this oxidative stress, where superoxide radicals react with hydrogen peroxide to produce hydroxyl radicals and additional reactive species, compounding the potential for more cellular damage [183,184].

The interplay between iron and ROS is a critical aspect of cellular metabolism and signaling. ISCs are integral to this interplay, acting as both redox-activity locations and sensors of cellular iron levels [172,173]. The biogenesis of ISCs involves a complex assembly process that requires various proteins, including frataxin and the cysteine desulfurase IscS, which utilizes sulfur from cysteine to form clusters [173,186]. Disruptions in ISC biogenesis can lead to impaired mitochondrial function and increased oxidative stress, as demonstrated in conditions like Friedreich's ataxia, where frataxin deficiency results in iron accumulation and oxidative damage [173,185,187].

Furthermore, the regulation of iron homeostasis is directly linked to cellular responses to oxidative stress. Iron regulatory proteins (IRPs) play a crucial role in sensing cellular iron levels and modulate the expression of genes involved in iron uptake, storage, and utilization [173,175]. The activity of IRPs is altered under conditions of oxidative stress, leading to changes in iron metabolism that can exacerbate or mitigate oxidative damage [173,175]. This regulatory network highlights the importance of maintaining iron homeostasis to prevent oxidative stress and its associated pathologies.

In summary, the redox chemistry of iron underscores its dual purpose as both a necessary cofactor in biological processes and a potential source of oxidative damage. The complex mechanisms governing iron-sulfur cluster biogenesis and iron homeostasis are vital for cellular health, and their dysregulation can potentially lead to significant pathophysiological consequences.

B) Thiol Groups and Redox Biology:

Thiol groups, characterized by the presence of a sulfhydryl (-SH) functional group, are critical components in biological systems, particularly in the context of protein structure and function. Cysteine and methionine residues are two amino acids that contain thiol groups, and they play pivotal roles in various biochemical processes. Cysteine, in particular, is one of the least abundant amino acids yet is frequently found in highly conserved regions of proteins, often at active sites where it is involved in catalytic and regulatory functions [188]. The unique reactivity of the thiol group allows cysteine to form disulfide bonds, which are essential for maintaining protein structure and stability under oxidative conditions [189]. Methionine, while less reactive than cysteine, also contributes to redox biology through its role in the synthesis of S-adenosylmethionine. S- adenosylmethionine is a key methyl donor in numerous biochemical reactions [190].

Glutathione (GSH), a tripeptide composed of glutamate, cysteine, and glycine, serves as a major cellular antioxidant, playing a crucial role in maintaining redox homeostasis within cells. GSH is involved in the detoxification of reactive oxygen species (ROS) and other free radicals, protecting cellular components from oxidative damage [191]. The thiol group of cysteine in GSH is particularly reactive, allowing it to participate in redox reactions that neutralize harmful oxidants [192]. Moreover, GSH can undergo various post-translational modifications, which involves the covalent attachment of GSH to cysteine residues in proteins. This modification can alter protein function, stability, and interactions, serving as a regulatory mechanism in cellular signaling pathways [193].

In addition to S-glutathionylation, S-nitrosylation and S-persulfidation are other significant post-translational modifications involving thiol groups. S-nitrosylation entails the addition of a nitric oxide (NO) moiety to the thiol group of cysteine, modulating protein activity and function [194]. S-nitrosylation has been implicated in various physiological processes, including vasodilation, neurotransmission, and the regulation of apoptosis [195]. Protein S-persulfidation is the process by which thiol groups in cysteine residues are converted into persulfides, playing a crucial role in protecting thiols from irreversible oxidative damage. This evolutionarily conserved post-translational modification is one of the primary mechanisms through which H_2_S exerts its signaling functions [32,49,196]. Thiols are highly susceptible to oxidation, rendering cysteine residues non-functional by converting thiols to sulfinic and sulfonic acid derivatives. S-persulfidation counters this by converting thiols to more stable persulfide moieties which are less prone to irreversible oxidation [33]. These more stable moieties can absorb and neutralize oxidative stress, preserving the functionality of the cysteine residue. Additionally, S-persulfidation is reversible enabling dynamic regulation of protein structure and function in response to changes in cellular redox environment [197].

The biochemical properties of thiols are largely dictated by their pKa values, which influence their reactivity and the formation of thiolate anions. The pKa of the thiol group in cysteine is approximately 8.5–9.0, making it a weak acid [198]. This property allows thiols to exist in both protonated and deprotonated forms under physiological conditions. The deprotonated form, known as the thiolate anion (RS^−^), is a potent nucleophile that can participate in various biochemical reactions, including nucleophilic attacks on electrophiles and the formation of disulfide bonds [188]. The ability of thiols to form thiolate anions is crucial for their role in enzyme catalysis, particularly in the active sites of enzymes such as GSH transferases, where thiolate formation is a key step in substrate conjugation [198].

Thiolate anions exhibit enhanced nucleophilic properties compared to their protonated counterparts, making them highly reactive in biochemical contexts. This reactivity is exploited in various biological processes, including the detoxification of electrophilic compounds and the modulation of protein function through thiol-disulfide exchange reactions [189]. The formation of disulfide bonds between thiol groups is a critical mechanism for stabilizing protein structures, an seen in extracellular environments where oxidative conditions prevail [188]. Furthermore, the dynamic nature of thiol chemistry allows for reversible modifications, enabling cells to respond to changes in their redox state and maintain homeostasis [192].

To summarize, thiol groups, particularly those found in cysteine and methionine, are integral to redox biology and cellular signaling. The unique biochemical properties of thiols, including their pKa values and the ability to form thiolate anions, are a driving force behind their importance in various biological processes. Understanding thiol chemistry is essential for piecing apart the mechanisms underlying cellular functions and the pathophysiology of diseases associated with redox dysregulation.

C) Iron-Thiol Interactions: Mechanisms and Pathophysiology:

**Formation of Iron-Thiol Complexes:** Iron-thiol interactions are pivotal in various biochemical processes, noticeably in the formation of iron-thiol complexes involving Fe^2+^ and Fe^3+^ ions with thiol-containing compounds such as cysteine, GSH, and protein thiols. The coordination of iron ions with thiols leads to the formation of stable complexes, which can significantly influence cellular redox states and protein functionality. For instance, the reaction of Fe^2+^ with thiols results in the formation of iron(III) thiolate complexes, which are crucial in biological catalysis and the stabilization of reactive intermediates [199,200]. The ability of thiols to coordinate with iron not only facilitates the formation of these complexes but also modulates the redox properties of iron, allowing for dynamic interplay between oxidation states that is essential for many metabolic pathways [201,202].

The interaction between iron and thiols is not merely a static binding event; it is a dynamic process that influences the structural integrity of proteins. Iron-sulfur clusters, which are integral to many enzymes, rely on the coordination of iron with thiol groups from cysteine residues. These clusters play a critical role in electron transfer and redox reactions within the cell [203]. The presence of thiols stabilizes the iron-sulfur cluster, enhancing the enzyme's catalytic efficiency and protecting it from oxidative damage [201,202]. Moreover, the formation of these complexes also influence the overall conformation of proteins, affecting their biological activity and interaction with other biomolecules [204].

The redox cycling of iron mediated by thiol groups is another critical aspect of iron-thiol interactions. Thiols can facilitate the reduction of ferric iron (Fe^3+^) to ferrous iron (Fe^2+^), a process that is vital for maintaining iron homeostasis in biological systems [205]. This reduction is particularly relevant in the context of oxidative stress, where the balance between reduced and oxidized forms of iron can dictate cellular responses to various stimuli, including inflammation and apoptosis [205,206]. The ability of thiols to modulate iron's oxidation state adds to their importance in cellular signaling and metabolic regulation.

**Oxidative Modifications Involving Iron and Thiols:** In addition to iron-thiol cluster formations, iron-thiol interactions also encompass oxidative modifications that can lead to significant biological consequences. One of the primary oxidative modifications involving iron is the oxidation of thiols, leading to the formation of disulfides. This process is often catalyzed by ROS generated in the presence of iron, which can oxidize thiol groups, resulting in the formation of disulfide bonds [205,207]. The formation of disulfides is a key regulatory mechanism in cellular signaling, as it can alter protein function and stability, impacting various physiological processes [205,207].

The thiol-driven reduction of ferric iron (Fe^3+^) is another crucial aspect of iron-thiol interactions. Thiols can effectively reduce Fe^3+^ back to Fe^2+^, which is needed for several biochemical reactions, including those involved in energy metabolism and antioxidant defense [205,206]. This reduction process not only helps maintain iron in its bioavailable form but also plays a role in mitigating oxidative stress by scavenging free radicals and preventing lipid peroxidation. The implications of this thiol-driven reduction extend to various pathophysiological conditions, including cancer, where altered redox states can influence tumor progression and response to therapy [205,207].

Moreover, the oxidative modifications induced by iron can lead to the generation of reactive thiol species, such as thiyl radicals (RS^•^), which can further participate in redox reactions and contribute to cellular signaling pathways [208]. The balance between thiol oxidation and reduction is crucial for maintaining cellular homeostasis, and disruptions in this balance can lead to pathological conditions, including neurodegenerative diseases and metabolic disorders [205,207]. Understanding the mechanisms underlying iron-thiol interactions and their oxidative modifications is essential for elucidating their roles in health and disease.

**Fig. 4:**
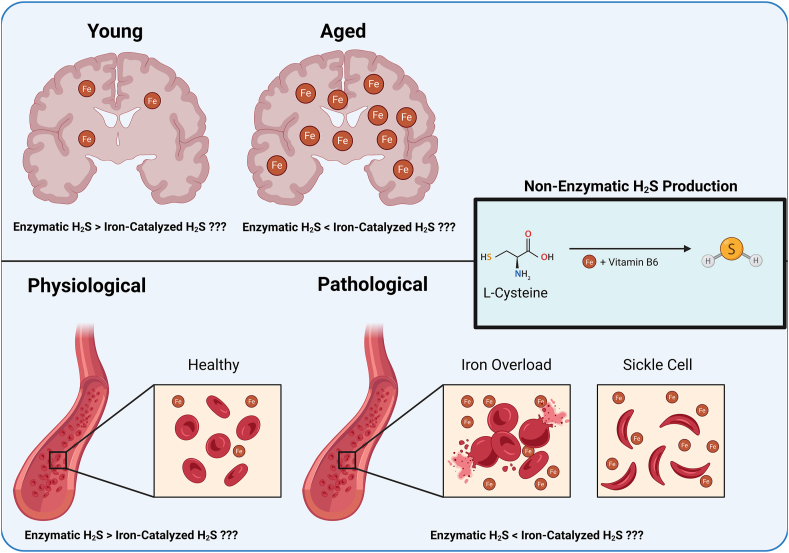
**Iron Dysregulation in the Brain and Circulation may Stimulate Under-Controlled Iron-Catalyzed Hydrogen Sulfide Production.** On the right is a basic overview of iron and vitamin B_6_ co-catalyzing H_2_S from the breakdown of cysteine. On the top depicts aging-related iron overload in the brain, and how this may lead to uncontrolled iron-catalyzed H_2_S production. On the bottom depicts iron dysregulation in the circulation either by hemochromatosis or sickle cell crisis and how this may lead to uncontrolled iron-catalyzed H_2_S production systemically.

More recently, we discovered the production of hydrogen sulfide (H_2_S) from cysteine can be catalyzed by iron in a non-enzymatic process under physiological conditions, demonstrating what while enzymatic production of H_2_S is inducible and robust in select tissues, the iron-catalyzed production contributes to the basal levels of H_2_S systemically. This non-enzymatic reaction, while relatively understudied at this time particularly *in vivo*, has several potential pathophysiological implications in various disorders including hemolytic, iron overload, and hemorrhagic conditions (Fig. 4). Systemically, these disorders and/or conditions include sickle cell anemia, hemochromatosis, and frequent blood transfusions [209], while in the brain iron and iron complexes buildup during aging in regions associated with motor and cognitive impairment [210]. More research will be needed to understand the consequences, importance, and implications of this non-enzymatically regulated reaction and the ability to harness it for therapeutic endpoints.

**Fig. 5:**
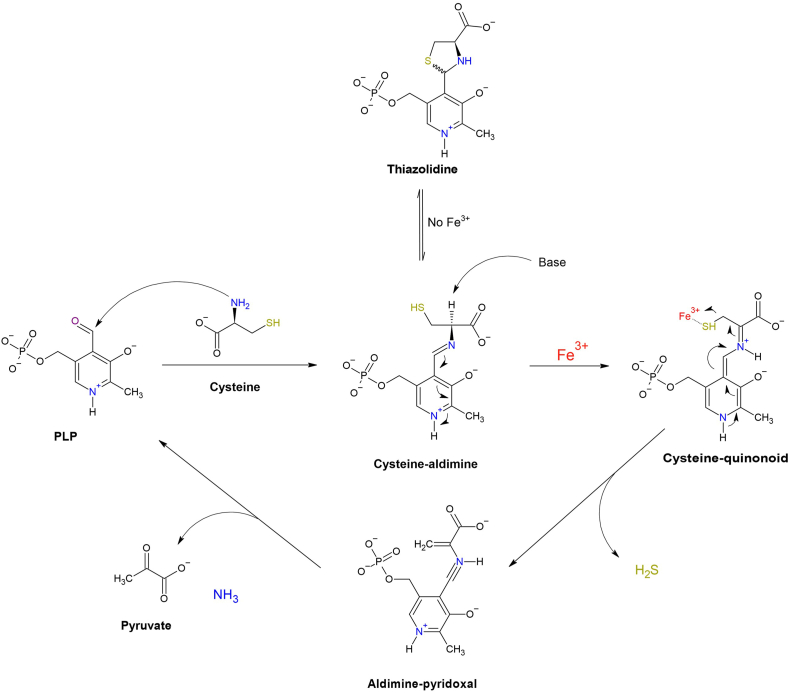
**Mechanistic Model for Iron and Vitamin B_6_-Catalyzed H_2_S Production from Cysteine.** The reaction includes a nucleophilic attack by the free amino group of cysteine on Vitamin B_6_ to form a Schiff base and subsequent cysteine-aldimine. Next, there is deprotonation at the α-position of cysteine leading to the formation of a quinonoid intermediate, where the elimination of –SH group is catalyzed by Iron III (Fe^3+^). Next, the desulfurated aldimine is hydrolyzed to produce pyruvate, NH_3_, and regenerate the Vitamin B_6_ co-factor. However, in the absence of iron, a 5-member thiazolidine ring is formed from the cysteine-aldimine product. This mechanisms and chemical model were adapted from Yang et al. [55].

This chemical reaction centers around the ultimate conversion of cysteine to pyruvate, ammonia, and H_2_S, with iron co-catalyzing the reaction along with Vitamin B_6_ in either its pryridoxal-5′-phosphate (PLP) or non-phosphate forms [46]. A brief overview of the proposed chemical mechanism is presented in Fig. 5.

D) Physiological Roles of Iron-Thiol Reactions:

**Cellular Iron Homeostasis and Redox Balance:** Iron-thiol interactions play a pivotal role in maintaining mitochondrial function, which is crucial for cellular energy production and overall cellular health. Mitochondria are the primary sites of aerobic respiration and are reliant on iron for the assembly of iron-sulfur (Fe–S) clusters, which are essential cofactors for various mitochondrial enzymes involved in the electron transport chain and Krebs cycle [211,212]. The delicate balance of iron within mitochondria is regulated by several proteins, including frataxin, acting as an iron chaperone. This ensures iron is available in a non-toxic form for enzymatic reactions while preventing oxidative damage [213,214]. Disruption in mitochondrial iron homeostasis can lead to mitochondrial dysfunction, which is implicated in various pathophysiological conditions, including neurodegenerative diseases and heart failure [215,216].

Moreover, the redox state of thiols within mitochondria is crucial for maintaining mitochondrial integrity and function. Thiol groups, particularly in GSH, serve as antioxidants that protect mitochondrial proteins from oxidative damage [217,218]. The interplay between iron and thiols is particularly significant; iron can catalyze the formation of reactive oxygen species (ROS), which can oxidize thiols, leading to a cascade of oxidative stress that compromises mitochondrial function [219,220]. Oxidative stress can result in the opening of the mitochondrial permeability transition pore, leading to cell death [221,222]. Therefore, the regulation of iron-thiol interactions is essential for preserving mitochondrial function and preventing oxidative damage.

In addition to their structural roles, iron-thiol interactions are also involved in cellular signaling pathways that regulate mitochondrial biogenesis and function. For instance, iron availability influences the activity of various transcription factors that promote the expression of genes involved in mitochondrial biogenesis [223,224]. The redox state of thiols can modulate the activity of these transcription factors, thereby linking iron metabolism with cellular signaling pathways that respond to oxidative stress [225]. This regulatory mechanism underscores the importance of iron-thiol chemistry in maintaining cellular redox balance and mitochondrial health.

**Fig. 6:**
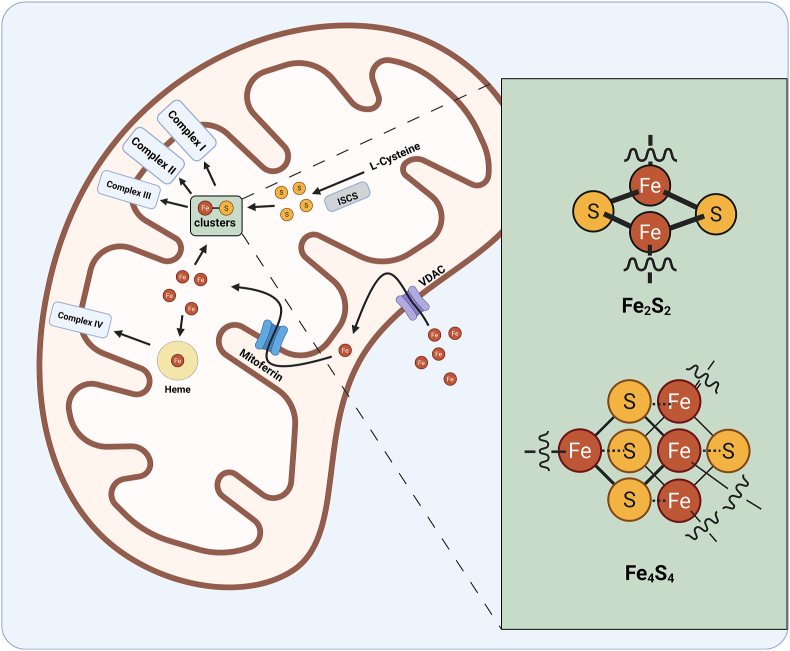
**Illustration of localized mitochondrial iron import and utilization in Fe–S clusters.** Iron enters the intermembrane space from the cytosol via voltage-dependent anion channels (VDACs) in the outer membrane. Iron is then transported into the matrix by mitoferrin. In the matrix, Fe–S cluster biogenesis and heme synthesis occurs. Fe–S clusters are then integrated into complexes I, II, and III for electron transfer while heme is utilized in complex IV.

Structurally, transfer of iron is facilitated by specific inner membrane transporters to the mitochondrial matrix such as mitoferrin. The process of iron-sulfur (Fe–S) cluster biogenesis then occurs within the mitochondrial matrix using ISC scaffolding [226]. Fe–S clusters created in this way then play direct roles in the function of mitochondrial complexes I, II, and III. Additionally, Fe–S clusters and Heme are generated from the same iron pool [226]. A broad overview of this sequence is illustrated in Fig. 6.

**Iron-Thiol Interactions in Enzymatic Activity:** Iron-thiol interactions are integral to the function of several key enzymes, including ribonucleotide reductase and aconitase. Ribonucleotide reductase, which is essential for DNA synthesis, requires iron for its activity. The enzyme's function is regulated by the availability of iron and the redox state of thiols, which can influence the enzyme's conformation and activity [227]. Similarly, aconitase, an enzyme involved in the Krebs cycle, contains an iron-sulfur cluster that is critical for its catalytic activity. The activity of aconitase is directly affected by iron availability; iron deficiency leads to decreased aconitase activity, which can disrupt cellular metabolism and energy production [228].

H_2_S has been implicated a modulator of ferroptosis. Ferroptosis is an iron-dependent form of regulated cell death characterized by the accumulation of lipid peroxides. H_2_S can inhibit ferroptosis by upregulating the expression of CGL, maintaining GSH levels and GPX4 activity. Conversely, downregulation of the CGL/H_2_S pathway has been shown to exacerbate ferroptosis [229,230]. H_2_S can interact with other signaling pathways to regulate ferroptosis. H_2_S has been reported to inhibit the activity of acyl-CoA synthetase long-chain family member 4 (ACSL4), an enzyme that promotes the synthesis of lipid peroxides and ferroptosis [229]. Furthermore, H_2_S can modulate the expression of ferroptosis-related genes, such as SLC7A11 and GPX4, which are crucial for maintaining intracellular GSH levels and preventing lipid peroxidation [231,232].

The modulation of enzyme activity via iron-thiol interactions is a finely tuned process. The oxidation of thiol groups can lead to the formation of disulfides, altering the conformation and activity of enzymes like aconitase [217,233]. This regulation is crucial for adapting to changes in cellular iron levels and oxidative stress. In conditions of iron overload, the excess iron can lead to the generation of ROS, further oxidizing thiols and impairing enzyme function [220,234]. Conversely, iron-deficient conditions can limit the formation of Fe–S clusters, leading to decreased enzyme activity and impaired metabolic processes [235].

The interaction between iron-thiol coordination and other cellular pathways is crucial for maintaining metabolic homeostasis. For instance, the thioredoxin and GSH systems regulate the redox state of thiols and modulate the activity of iron-dependent enzymes by working in tandem [225,236]. Crosstalk between different redox systems highlights the complexity of iron-thiol interactions in cellular metabolism and signaling. Disruptions in these interactions potentially lead to pathological conditions, emphasizing the importance of iron-thiol homeostasis for optimal cellular function [212].

E) Pathophysiological Implications of Iron-Thiol Dysregulation:

Iron overload and Oxidative Stress: Iron overload plays a crucial role in several pathophysiological conditions, particularly in hematological disorders. The labile iron pool (LIP) promotes thiol oxidation and the production or ROS. The LIP consists of non-transferrin-bound iron and is highly reactive, catalyzing the Fenton and Haber-Weiss reactions [237,238]. The generated ROS can cause extensive damage to cellular components such as lipids, proteins, and DNA, contributing to oxidative stress and cellular dysfunction [237,239]. This can eventually lead to tissue damage and organ failure, as shown in hemochromatosis [240,241].

The physiological mechanisms by which iron induces cellular damage are complex and often multifaceted. For example, a common treatment for severe thalassemia is blood transfusions. However, this can lead to iron overload and is compounded by already present ineffective erythropoiesis and low hepcidin levels [242,243]. The oxidative stress caused by the iron overload can trigger apoptosis and necrosis in various cell types leading to cardiovascular complications like cardiomyopathy and diabetes for reasons mentioned below [244,245]. Accumulation of iron in the myocardium is of particular concern, as it can lead to heart failure and cardiac hypertrophy. These are both frequently observes in patients with hemochromatosis and thalassemia [246,247].

**Iron-Thiol Dysregulation in Neurodegenerative Diseases:** Excess iron accumulation and thiol oxidation are contributors to the pathophysiology of neurodegenerative diseases, such as Parkinson's and Alzheimer's. In these conditions, thiol oxidation is promoted by elevated levels of iron and lead to the formation of protein aggregates. These are a common characteristic of neurodegenerative processes [248,249]. In particular, the interaction between iron and GSH is critical as it modulates the redox states of neurons and can contribute to neuronal cell death [250,251]. In Parkison's disease, iron-induced oxidative stress leads to the aggregation of alpha-synuclein. This is a hallmark of the disease and exacerbates the neurodegeneration [248,251].

In terms of mechanism for persulfidation, this rests on its ability to prevent irreversible oxidative damage to proteins, as well as potential alterations in structure and function of a protein. Persulfidation of and subsequent inhibition of S-adenosylhomocysteine hydrolase (SAHH) at Cysteine 195 can induce ferroptosis in NSCLC cells [252]. Inhibition of SAHH lead to a decrease in homocysteine and GSH resulting in ferroptosis. In general, a global decrease of protein persulfidation has been observed for several neurodegenerative diseases [115]. However, persulfidation of specific proteins have been observed and studied. Parkin, an E3 ubiquitin ligase with reactive thiol groups, can be persulfidated at Cysteine 59, 95, and 182, resulting in increased enzyme activation and clearance of damaged proteins. Samples from Parkinson's disease patients have been found to contain lower levels of persulfidated parkin, supporting persulfidation's protective role in a neurodegenerative setting [253].

Transferrin-bound iron is typically regulated and is safely transported in plasma to cells as needed [254]. Conversely, non-transferrin bound iron is a redox-active species prone to the catalyzation of ROS via the Fenton reaction and generally indicates a state of iron overload. This promotes a positive feedback loop where excess oxidative stress caused by iron leads to the production of more iron accumulation, further increasing oxidative stress [255]. This eventually leads to neurotoxicity or cell death via ferroptosis [256]. These examples highlight the need for iron hemostasis to mitigate the risk and severity of neurodegenerative diseases.

**Iron-Thiol Interactions in Cardiovascular Diseases:** Iron-catalyzed thiol oxidation has an important role in the pathogenesis of cardiovascular diseases, particularly in the context of endothelial dysfunction and atherosclerosis. The oxidative modification of thiols can impair endothelial function, leading to increased vascular permeability and inflammation. In atherosclerosis, the excess accumulation of iron in macrophages promote the formation of foam cells [257,258]. Foam cells are lipid-laden cells containing cholesterol and contribute to plaque development. Additionally, the presence of oxidized lipids exacerbates the inflammatory response.

Iron overload has been implicated in platelet activation and thrombosis, exhibiting a complex interaction that both inhibits and increases platelet reactivity depending on the conditions. Iron overload provides the formation of ROS, which can enhance platelet activation and aggregation particularly in patients who have undergone multiple transfusions [[257], [258], [259], [260]]. However, excess iron saturates transferrin and been associated with platelet inhibition as iron transferrin saturation increases [[261], [262], [263]]. Thus, understanding the dynamics of iron-thiol interactions is crucial for developing therapeutic strategies aimed at preventing cardiovascular complications associated with iron overload.

**Iron-Thiol Reactions in Cancer and Metabolic Diseases**: Abnormal iron metabolism and thiol oxidation have increasingly been recognized as important factors in tumorigenesis. In cancer, the dysregulation of iron homeostasis can lead to increased oxidative stress, promoting DNA damage and genomic instability [250,264]. Iron and thiol reactions, particularly when GSH is involved, are crucial to maintaining cellular redox balance homeostasis and preventing tumor progression. Elevated levels of ferritin and transferrin saturation have been correlated with poorer prognoses in various cancers, emphasizing the role of iron-thiol in cancer biology [250,264].

The role of GSH-iron interactions in chemotherapy resistance is a growing fiend of research. Several chemotherapeutics rely on the generation of ROS to induce cell death. However, cancer cells often exhibit dysregulated iron metabolism and higher levels of GSH, enhancing antioxidant capacity and resistance to chemotherapy [[265], [266], [267]].

**Iron-thiols in Aging:** The dysregulation of iron homeostasis has been implicated in the pathogenesis of several age-related diseases and neurodegenerative disorders [[268], [269], [270], [271]]. As individuals age, there is a well-documented accumulation of non-heme iron in various brain regions, which can lead to increased oxidative stress and cellular damage [[272], [273], [274], [275]]. This iron overload has been linked to the development of age-related neurodegenerative diseases, such as Alzheimer's and Parkinson's disease [271,276,277]. The underlying mechanisms involve the catalysis of Fenton reactions, leading to the production of reactive oxygen species (ROS) and subsequent lipid peroxidation, protein oxidation, and cellular dysfunction [269,278,279].

During the aging process, there is often a depletion of GSH and an increase in ferrous iron, which can lead to the activation of ferroptosis [[280], [281], [282]]. Furthermore, the regulation of iron homeostasis is a complex process involving various proteins and signaling pathways, such as hepcidin, transferrin, and ceruloplasmin [[283], [284], [285], [286]]. Dysregulation of these iron-regulatory mechanisms has been observed during aging and can contribute to the accumulation of iron in various tissues, including the brain, retina, and skeletal muscle [269,[287], [288], [289]].

To summarize, the dysregulation of iron-thiol interactions has profound implications across various pathophysiological conditions, including oxidative stress, neurodegeneration, cardiovascular diseases, and cancer. Understanding these interactions is essential for developing targeted therapeutic strategies aimed at mitigating the detrimental effects of iron overload and improving patient outcomes.

F) Therapeutic Strategies Targeting Iron-Thiol Interactions:

**Antioxidant Therapies:** The therapeutic landscape for managing oxidative stress and iron overload has increasingly focused on thiol-based antioxidants, such as N-acetylcysteine (NAC) and glutathione (GSH). These compounds play a role in maintaining redox homeostasis by neutralizing ROS and restoring thiol levels in pathological conditions. For example, NAC has been demonstrated to restore thiol levels and reduce oxidative stress in conditions such as cystic fibrosis and other inflammatory diseases [290,291] However, the application of thiol-based antioxidants is always approached with extreme caution, as excessive modulation of thiol redox status can lead to adverse effects, including disruption of signaling pathways dependent on disulfide bonds [292],sulfur amino acid sensing [74], and proper adaptive responses to exercise and dietary restriction [9,74].

Moreover, the interplay between iron and thiol antioxidants is complex. Studies have indicated that iron loading can alter the balance between reduced GSH and its oxidized form (GSSG), leading to increased oxidative stress [293]. This suggests that while thiol-based antioxidants can mitigate oxidative damage, their efficacy may be compromised in the presence of elevated iron levels. Therefore, the strategic use of thiol antioxidants in conjunction with iron chelation therapies may enhance therapeutic outcomes by both addressing oxidative stress and iron overload.

Iron chelators, such as deferoxamine and deferasirox, have emerged as critical components in the management of iron-related disorders. These agents work by binding free iron, thus preventing its participation in Fenton reactions that generate harmful hydroxyl radicals [294,295]. Deferasirox has shown promise in clinical settings, demonstrating a stronger chelation ability compared to deferoxamine, which may enhance its effectiveness in reducing iron-induced oxidative damage [294]. The combination of thiol-based antioxidants with iron chelation strategies represents a promising avenue for therapeutic intervention, particularly in conditions characterized by both oxidative stress and iron overload.

Despite potential promising advancements and theories in the use of antioxidants, thiol-based therapies, iron-chelators, and/or iron-thiol targeted antioxidants in a clinical setting, significant hurdles remain that call into question of consistency and clinical applicability. This primarily stems from an oversimplistic understanding of ROS and antioxidants in maintaining homeostasis. Although viewed in a negative context, ROS remains essential for cellular signaling and requires highly specific and targeted modulation to avoid disrupting normal physiology [296,297]. However, most antioxidants lack target specificity, making it difficult to modulate pathological oxidative stress without disrupting essential redox processes. Heterogeneity of disease states and appropriate dosing is complicated as some antioxidants are shown to become toxic metabolites at high doses. Additionally, it has been demonstrated that intracellular antioxidants like GSH behave differently from supplementation, questioning the reliability of supplement-based antioxidant trials [298]. Addressing these concerns is crucial for effective consistent clinical use of antioxidant therapy.

**Modulation of Iron Homeostasis**: Regulating iron homeostasis is crucial for mitigating the detrimental effects of iron overload and oxidative stress. Therapeutic approaches aimed at modulating iron levels include the use of iron chelators, as well as the modulation of hepcidin, a key regulator of iron metabolism. Hepcidin functions by inhibiting ferroportin, the primary iron export protein, thereby reducing iron absorption from the diet and iron release from macrophages [299,300]. Recent studies have highlighted the potential of hepcidin modulation as a therapeutic strategy for various iron-related disorders, including anemia of chronic disease and hereditary hemochromatosis [301,302].

Intermittent fasting has also been proposed as a non-pharmacological intervention to restore redox balance and regulate iron metabolism. This dietary approach may enhance the body's endogenous antioxidant defenses while simultaneously reducing iron absorption [303]. Additionally, sulfide donors have garnered attention for their potential to modulate iron homeostasis and exert protective effects against oxidative stress. H_2_S has been shown to influence iron metabolism by promoting the expression of hepcidin and reducing iron overload in various models [304].

The integration of these therapeutic strategies—iron chelation, hepcidin modulation, and dietary interventions—offers a multifaceted approach to restoring iron homeostasis and mitigating oxidative stress. By targeting both iron metabolism and redox balance, these strategies hold promise for improving outcomes in a range of pathophysiological conditions, including neurodegenerative diseases, cancer, and inflammatory disorders [305,306].

Interestingly, lifestyle factors, such as CR and exercise, have been shown to modulate iron metabolism and potentially mitigate the adverse effects of age-related iron accumulation [307,308]. Additionally, pharmacological interventions targeting iron chelation or the modulation of iron-related signaling pathways have been explored as potential therapeutic strategies for age-related diseases [210,309,310].

Therapeutic strategies targeting iron-thiol interactions encompass a diverse array of approaches aimed at mitigating oxidative stress and restoring iron homeostasis. The use of thiol-based antioxidants, in conjunction with iron chelation therapies, presents a synergistic strategy for addressing the complexities of iron overload and oxidative damage. Furthermore, the modulation of iron metabolism through hepcidin regulation and dietary interventions such as intermittent fasting and H_2_S donors provides additional avenues for therapeutic exploration. As research continues to investigate the intricate relationships between iron, thiols, and oxidative stress, the development of targeted therapies may lead to significant advancements in the management of many pathophysiological conditions.

## Discussion

Iron and sulfur are unique in their ability to exist in multiple redox states, a property that underpins their essential role in numerous biochemical processes. This versatility is especially evident in the dynamic interplay between iron and thiols, which orchestrates a range of critical physiological functions and safeguards cellular homeostasis. Iron-thiol interactions are central to maintaining the delicate balance of redox biology, underscoring their importance in normal cellular physiology and their potential as a therapeutic target in disease states.

The intricate biochemistry of iron, thiols, and H_2_S represents a cornerstone of redox biology, with profound implications for both physiological function and disease such as neurodegeneration and glioblastoma. In this review we discussed how in the processes of normal and pathological aging, there are imbalances in iron, thiol, and H_2_S homeostasis. Whether they are causative or merely correlative is to be debated, but a good deal of evidence leans toward the former and not the latter. However, much is to be done to bring geroscience approaches to the clinical to regain H_2_S and iron-thiol homeostasis during aging, and that journey relies on the field probing for deeper understandings of cell- and tissue-specific responses to H_2_S. By targeting and harnessing the delicate balance of H_2_S being a therapeutic vs. toxin, the promise of mitigating the pathophysiological consequences of redox imbalance and thus enhancing healthspan will be within reach.

## Disclosure statement

C.H. is a consultant to Treasure Biosciences, a company developing interventions for ovarian aging. The content of this article was not influenced by Treasure Biosciences nor does it relate to ovarian aging.

## Author contributions

M.G. and C.H. conceived of the review article and wrote the article. Both authors edited and revised the article and composed the figures.

## Declaration of competing interest

The authors declare the following financial interests/personal relationships which may be considered as potential competing interests: Christopher Hine reports a relationship with Treasure Biosciences that includes: consulting or advisory and equity or stocks. If there are other authors, they declare that they have no known competing financial interests or personal relationships that could have appeared to influence the work reported in this paper.
